# Men’s Attitudes Towards Participation in Organised Prostate Cancer Testing: An Abductive Thematic Analysis

**DOI:** 10.1016/j.euros.2024.12.007

**Published:** 2024-12-27

**Authors:** Markus Arvendell, Lottie Phillips, Sara Delilovic, Moa Backman Enelius, Karin Olsson, Anetta Bolejko, Olof Akre, Sigrid Carlsson, Anne Richter, Anna Lantz

**Affiliations:** a Department of Molecular Medicine and Surgery, Karolinska Institutet, Stockholm, Sweden; b Department of Medical Epidemiology and Biostatistics, Karolinska Institutet, Stockholm, Sweden; c Department of Urology, Södersjukhuset, Stockholm, Sweden; d Centre for Epidemiology and Community Medicine, Region Stockholm, Stockholm, Sweden; e Department of Learning, Informatics, Management and Ethics, Karolinska Institutet, Stockholm, Sweden; f Department of Translational Medicine, Lund University, Malmö, Sweden; g Department of Medical Imaging and Physiology, Skåne University Hospital, Malmö, Sweden; h Departments of Surgery (Urology Service) and Epidemiology and Biostatistics, Memorial Sloan Kettering Cancer Center, New York, NY, USA; i Department of Urology, Institute of Clinical Sciences, Sahlgrenska Academy, University of Gothenburg, Gothenburg, Sweden; j Department of Translational Medicine, Division of Urological Cancers, Medical Faculty, Lund University, Lund, Sweden

**Keywords:** Prostate cancer, Organised testing, Attitudes, Abductive thematic analysis, Qualitative study

## Abstract

**Background and objective:**

Organised prostate cancer (PCa) testing (OPT) was introduced in Sweden to gain knowledge in preparation for a potential national PCa screening programme. This study aims to explore men’s opinions regarding the OPT invitation letters and the attitudes influencing their decision to participate in or decline OPT.

**Methods:**

We conducted semi-structured telephone interviews with 30 men (nine participants and 21 non-participants) from Stockholm County who received OPT invitations. We employed an abductive thematic analysis, a reflexive process of identifying theoretical explanations of emerging patterns, to identify themes in men’s responses.

**Key findings and limitations:**

Informants found the invitation letters informative and appreciated the screening opportunity, but suggested improvements regarding conciseness and clarity about the risks and benefits of testing. Barriers to participation included lack of time or motivation, fear of discovering illness, inaccessibility, and distrust of health care or medical procedures. Facilitators included a desire to confirm or rule out PCa, and taking advantage of the available screening opportunity. Limitations include the study’s single-county focus and a potential recall bias affecting responses.

**Conclusions and clinical implications:**

Men’s attitudes towards OPT participation are multifaceted. While men appreciate screening opportunities, practical considerations as well as personal and psychological factors influence their participation decisions. To improve informed decision-making, OPT communication should be clear about the benefits and risks, and accessibility and logistical challenges should be addressed. Enhancing understanding and reducing fears are essential for refining screening practices and aligning these with men’s needs.

**Patient summary:**

Men value the option for prostate cancer screening, but face barriers such as low motivation and fear of a cancer diagnosis. Clear communication could improve their understanding of screening and encourage informed decision-making regarding participation in organised prostate cancer testing. Improved accessibility to testing could enhance participant opportunity.

## Take Home Message

Men appreciate prostate cancer screening opportunities, but their participation is influenced by practical, personal, and psychological factors. Clear communication about the benefits and risks of testing, as well as addressing logistical barriers, can enhance informed decision-making and improve screening practices.

## 1 Introduction

Screening with prostate-specific antigen (PSA) combined with systematic prostate biopsies reduces prostate cancer (PCa) mortality [1], [2], [3], [4] but poses risks of overdiagnosis and overtreatment of clinically insignificant tumours [5], [6]. Consequently, PSA testing guidelines vary internationally, with most recommending individualised, shared decision-making [7], [8], [9]. Recent advancements in prostate magnetic resonance imaging (MRI) before biopsy may reduce the detection of low-risk PCa and mitigate overdiagnosis, reigniting discussions on population-based PCa screening strategies [10], [11], [12], [13]. In 2018, the Swedish National Board of Health and Welfare advised against PSA screening, but directed regional cancer centres to develop standardised testing protocols to gain knowledge in preparation for a potential national PCa screening programme. This resulted in population-based regional projects known as organised prostate cancer testing (OPT), which began in 2020 and are now active in most Swedish regions [14], [15].

OPT combines PSA, MRI, and biopsies using specific algorithms [16]. In Stockholm County, the process begins with two invitation letters: one explaining the benefits and risks of early PCa detection, and the other offering a free PSA test within 4 weeks of receipt [14]. Participation is voluntary, and little is known about the factors influencing men’s participation decisions.

Few studies have explored men’s attitudes towards PCa screening, with most focusing on non-European populations. Existing research from Saudi Arabia, Zimbabwe, Tanzania, Jamaica, Australia, Nigeria, and Ireland shows that men have limited PCa knowledge and varied attitudes towards screening, but does not clearly link these attitudes to participation decisions [17], [18], [19], [20], [21], [22], [23]. A systematic review of qualitative studies found that men were willing to undergo testing for cancer prevention and reassurance, especially when encouraged by social networks or health care providers. However, they had to overcome fears of losing masculinity, screening intrusiveness, and doubts about its necessity or costs [24]. Some men chose PSA testing due to beliefs in early diagnosis, often influenced by personal experiences, family history, media reports, or urinary symptoms [25]. Others avoided testing due to a perceived low risk from the absence of symptoms or family history, a belief that healthy behaviours could prevent cancer, or feelings of fear, embarrassment, or scepticism about screening [26]. Since organised testing is scarcely implemented, focus has been on opportunistic testing using shared decision-making. A survey-based OPT study found that most men appreciated the testing opportunity [27]. However, participation rates in PCa screening trials have varied. The large European Randomised Study of Screening for Prostate Cancer (ERSPC) trial achieved a high participation rate of around 80% [28], while the contemporary STHLM3 trial reported about 40% [29]. In the first just over 2 years of the OPT programme, participation reached 35% [30]. The reasons for these relatively low participation rates in the OPT programme are unclear, and high-quality evidence on the factors influencing men’s decisions to engage in organised testing is lacking. Understanding these factors will be essential for the potential implementation of screening programmes in the future.

This study aims to explore men’s opinions on the information provided in the two invitation letters and the attitudes influencing their decision to participate in or decline OPT.

## 2 Methods

### 2.1 Study design

We conducted semi-structured telephone interviews from April to June 2023, interviewing each informant within 2 months of their OPT invitation. The invitation involved two letters, mailed separately and 2 weeks apart. The first letter was a preparatory letter, briefly explaining the benefits of testing (early PCa detection) and the risks (detection of nonsignificant PCa and potential adverse effects from PCa treatment), while also notifying recipients of an upcoming testing offer. The second letter included a PSA testing offer, available within 4 weeks of receipt. Both letters contained a QR code for accessing translations in seven languages, and the second letter also provided a QR code with the locations of approximately 40 testing sites across Stockholm County. The decision to send two separate letters was made by the Regional Cancer Centre in Stockholm County to facilitate participation.

Recruitment for the interviews was based on personal identification numbers and residential addresses from the OPT-IT registry. Informants were recruited through two methods: phone numbers either obtained from online directories based on residential addresses or provided through a concurrent survey, as our data collection was part of a broader OPT evaluation project. The target group included men born in 1973 (aged 50 years at the time of data collection) and residing in Stockholm County, as the initial phase of OPT invited only men aged 50 years.

Ethical approval was granted by the Swedish Ethical Review Authority (DNR 2022-05079-01).

### 2.2 Data collection

We used purposive sampling, initially targeting men from low-participation municipalities (20–29% participation rates in OPT) and later expanding to include those from areas with medium (30–39%) and high (>40%) participation rates. Men were approached randomly within each group (low, medium, and high participation) without prior knowledge of their participation in OPT. We applied this strategy to include both participants and non-participants, as well as to cover the spectrum of attitudes and opinions from areas with varying participation rates. Initially, we prioritised men from low-participation areas, assuming that they would be more challenging to reach. As more informants agreed to be interviewed, we expanded to include men from medium- and high-participation areas, who were more likely to have participated in OPT. Our aim was to gather sufficiently detailed insights through in-depth interviews, estimating a total of 30 based on qualitative study design recommendations [31], [32].

A semi-structured interview guide was developed by the study team, covering questions about the content, distribution, and design of the letters; participation decisions; previous test history; reasons for their decision; impact of the letters; and other influencing factors. Interviews were recorded with informants’ consent for verbatim transcription. Written study information was provided via e-mail, and verbal consent was obtained and recorded before each interview began. The interviews were conducted by three study team members: a PhD student who is also a urology resident and two research assistants.

### 2.3 Analysis

An abductive thematic analysis was used to identify patterns in the data through a reflexive process of generating theoretical explanations for emerging themes [33], [34]. The analysis followed four stages. First, we reviewed the transcripts thoroughly to understand informants’ responses and their “thinking out loud” reflections. Second, relevant quotations were coded according to the study’s research questions using a deductive approach to (non-)participation. Third, we inductively reviewed the quotations to identify themes and subthemes representing concepts, similarities, and differences in informants’ attitudes and opinions. Finally, we defined and named the themes and subthemes to reflect informants’ views on (non-)participation in OPT. Some quotations were edited for clarity without altering their meaning.

## 3 Results

### 3.1 Informants

**Fig. 1:**
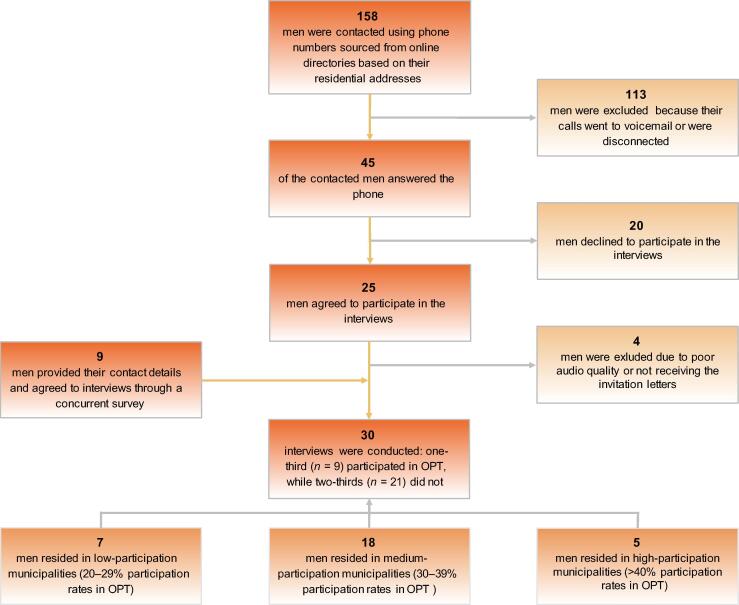
Flowchart of the inclusion process for interviewed men. *n* = number of individuals; OPT = organised prostate cancer testing.

Fig 1 illustrates the inclusion process for interviewed men. We conducted 30 interviews: seven from low-participation municipalities, 18 from medium-participation areas, and five from high-participation areas. Approximately one-third (*n* = 9) participated in OPT, while two-thirds (*n* = 21) did not. Among participants, one was from a low-participation area, five from medium-participation areas, and three from high-participation areas. Among non-participants, six were from low-participation areas, 13 from medium-participation areas, and two from high-participation areas.

### 3.2 Opinions on the invitation letters

Table 1 presents the identified themes from the interviews with corresponding quotations. Informants commonly reported that the letters reminded them of ageing, especially when received near their birthdays. Many expressed gratitude for the availability of screening opportunities in Sweden.

**Table 1:** Identified themes on letter content, distribution, and design, and corresponding quotations

Themes	Quotations (informant)
First impressions	“My goodness, am I really that old?” [24]
	“I received the letters just a week before I turned 50, which was a funny reminder of getting older.” [16]
	“You feel grateful, realising how fortunate we actually are.” [3]
Information load	“It didn’t feel like there were too many pages or long texts to get to the point.” [20]
	“People live a busy life, so the person writing doesn't get many seconds of attention.” [24]
Information clarity	“[Should] highlight early detection benefits.” [18]
	“It might be good to explain why you're aiming to screen people in general [...] and that it's not just one's opinion but statistically beneficial.” [6]
	“I was surprised they mentioned disadvantages since I primarily see the advantages of preventing cancer”. [4]
	“I would like a grading of the likelihood of experiencing a disadvantage. For example, what percentage of those tested end up undergoing unnecessary treatment? If there's a 50% risk of unnecessary treatment, you might opt not to get tested. But if the risk is only 0.5%, you'd likely choose to get tested. The letters emphasised disadvantages so much that it seemed the risk was quite high.” [1]
	“It was a long, extensive study [...], with several follow-ups. I remember it was written that I had to go to Karolinska Institutet and get tested, and then I felt, how exhausting, do I have to go there once or several times a month? No, I can't handle that.” [17]
Opinions on the use of two letters	“[Suggesting only one letter] Then you can see everything at once instead of in two rounds, and it's better for the environment too.” [16]
	“It felt a bit nagging but didn't affect my decision not to go while the offer was valid.” [5]
	“It gave me a heads-up about what was coming.” [13]
	“It gave more time to prepare, so it was excellent.” [17]
Opinions on the use of a QR code	“I think I did use it, but there was nothing new there, everything was already described [in the letters].” [5]
	“My camera doesn't have that feature, so I can't easily scan a QR code without an app. […] It wasn't clear where to go for testing [...] I was unsure if there was a specific place or if I could go to any of them.” [1]
	“An alternative to the QR code would have been preferable.” [5]
Layout and delivery method	“I remember it was really messy.” [14]
	“It was better to send a letter than an e-mail. E-mails tend to disappear. So, it felt quite serious.” [17]
	“It might be better if you get a text message [instead of a letter]. You can get quite a lot of mail, and then, you might forget about it.” [9]
	“Letters feel a bit old-fashioned, to be honest. I never check the mailbox, only my wife does.” [19]

Informants generally found the information provided to be adequate. While most considered the length appropriate, some preferred a more concise format due to busy lifestyles. Many felt that the letters did not clearly emphasise the benefits of testing or adequately explain the drawbacks, and some sought clearer information on the risks versus benefits of testing, including specific statistics on unnecessary treatments. There were also misunderstandings about the nature of OPT, with some mistakenly believing that it required extensive research participation.

Most informants had no strong opinions about receiving the OPT invitation in two separate mailings, and some were unsure whether they had received both. Many deemed it unnecessary and preferred a single letter for clarity and environmental considerations. A few found the two letters redundant, while others appreciated the advance notice and additional preparation time.

Informants who used the QR code to access testing site locations found it useful. None used it for translation, and few recalled using it for additional test information. One informant had difficulty scanning the code and locating the correct site.

Most informants had no strong opinions on the letter layout but suggested improvements, such as highlighting the pros and cons and clarifying blood testing instructions. Regarding delivery, most preferred the use of letters over potential e-mails for their formality and reliability. However, some felt that letters might be forgotten easily and recommended more immediate methods, such as text messages, for more effective reminders.

### 3.3 Attitudes towards OPT participation

**Fig. 2:**
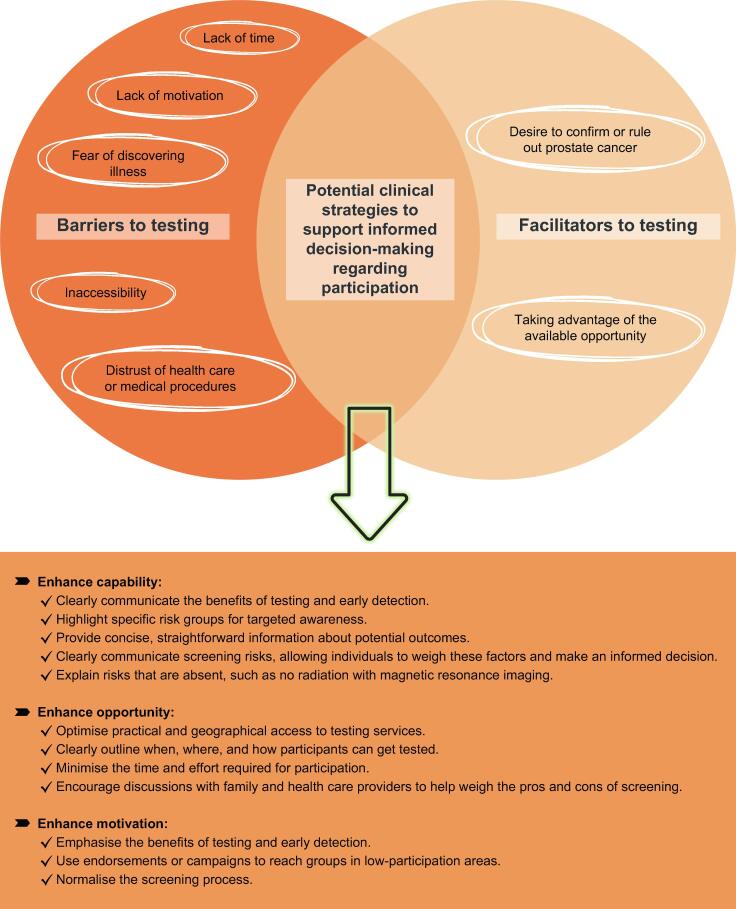
Identified themes and subthemes on barriers and facilitators to testing and potential clinical strategies to support informed decision-making regarding participation.

Two main themes emerged regarding men’s attitudes towards OPT participation: barriers and facilitators to testing. We identified five subthemes as barriers and two as facilitators (see Fig 2). Barriers included lack of time, lack of motivation, fear of discovering illness, inaccessibility, and distrust of health care or medical procedures. Facilitators included a desire to confirm or rule out PCa and taking advantage of the available opportunity.

#### 3.3.1 Barriers to testing

Table 2 presents the identified barriers to testing with corresponding quotations. A common barrier was a lack of time or inconvenient timing of the OPT invitation. Many struggled to prioritise testing due to busy schedules, with some considering the 4-week testing window too short, resulting in missed opportunities.

**Table 2:** Identified themes and subthemes on barriers and facilitators to testing, and corresponding quotations

Themes and subthemes	Quotations (informant)
Barriers to testing	
Lack of time	“No, I did not [regarding whether he was tested or not]. But it was mostly because I felt I didn't have the time.” [1]
	“It was during that period that I was away. So, my plan was to call when I got home and see if I could book a later appointment. But it didn't happen.” [2]
	“It's just poor prioritisation, I don't have a good answer. I work a lot, run several businesses, and have long days and so on. So, it's hard to schedule and, well, prioritise yourself, simply.” [3]
	“When I finally thought about getting tested, I realised that the time was up.” [5]
	“It was a rather short time to take the test. Two weeks might seem long, but it isn't. First, you need to handle the letter and think it over. People don't act immediately; they often deliberate and go back and forth.” [6]
Lack of motivation	“It's just about stopping the procrastination and start acting.” [8]
	“I thought about going because it's good [to detect potential illness] but then it was the combination of having other things to do, plus a bit of laziness.” [9]
	“We live in a country where they send out [offers for testing] and then you're so damn stupid that you don't go.” [3]
Fear of discovering illness	“My dad had prostate cancer, and my mom passed away from breast cancer. It feels overwhelming to face the possibility of having cancer myself. It's a sensitive topic, and people are simply afraid.” [11]
	“How should I put it? Rationally I understand that it's good to address any problems early if they exist. But there's also a part of me that prefers not to know. It's nice to be ignorant too.” [1]
Inaccessibility	“I live on the outskirts of the county, and the nearest testing facility is in the opposite direction. I simply couldn't find the time.” [13]
	“Just taking a blood test. You give up, I mean, if you live like I do. You have no idea where to go or how to proceed.” [14]
	“The information that I received was that you had to use a QR code, and the only reason I didn't participate [in OPT] was because I didn't have a QR code reader.” [15]
Distrust of health care or medical procedures	“Even if you get sick, you don't get help. [...] I don't want to waste my time.” [9]
	“It's very hard to know how, what you should do [...] Who you should trust, and so on.” [11]
	“Generally, I'm opposed to radiation, vaccines, and similar things [...] In retrospect, maybe some of these tests can be done with only blood samples, but if it involves x-rays and such, I don't want to expose myself to those forms of radiation.” [16]
Facilitators to testing	
Desire to confirm or rule out prostate cancer	“I saw only benefits in getting tested. It's better to know than not know. [...] I wanted to determine as soon as possible if I had prostate cancer so I could do something about it.” [18]
	“I have lost some friends and family members to cancer, so it's something you think about. It's good to detect it as early as possible.” [19]
	“I chose to focus on the benefits of monitoring this, as I've observed from close ones and relatives that cancer runs in the family.” [20]
	“My father got prostate cancer and discovered it relatively early due to a health check. His treatment went well, and it served as a wake-up call for me. I realised that any opportunity to monitor my own health should be taken.” [21]
Taking advantage of the available opportunity	“I hadn't considered it before, but when the offer came, I realised it was a great opportunity that I should definitely take advantage of.” [22]
	“I saw it as a small nudge in the right direction.” [20]
	“Otherwise, you might delay for 6 months or a year, as people often do with non-urgent matters. This felt important and timely. I thought, 'Perfect, it's time to go.” [23]

Lack of motivation, though not always stated explicitly, was also evident among non-participants. Competing priorities, such as personal interests or busy schedules, often took precedence, leading some to overlook the testing offer despite viewing it positively. In some cases, lack of time and low motivation collectively prevented participation, leading many to regret missing the opportunity.

Fear of discovering illness was also an identified barrier. Some men, particularly those with a family history of cancer, avoided testing due to anxiety about a potential cancer diagnosis. Others preferred to remain unaware of potential health issues and downplayed their risk, considering it to be minimal due to their age. Despite recognising the benefits of early detection, psychological discomfort in confronting illness deterred participation.

Physical and technical inaccessibility also posed challenges. Some men found the distance to the nearest testing site prohibitive, with one informant struggling to accommodate long travel times in his schedule. Technical inaccessibility, such as the need for a QR code to access information, also hindered participation, as one man lacked a QR code reader.

Distrust of health care or medical procedures also deterred some from testing. Negative prior experiences led one informant to question the value of seeking care, while another was concerned about and misunderstood the potential radiation exposure from scans.

#### 3.3.2 Facilitators to testing

Table 2 presents the identified facilitators to testing with corresponding quotations. The main facilitator was the desire to confirm or rule out PCa. Many men who had previously contemplating testing took advantage of the available opportunity. Influences included personal or familial cancer experiences, which increased their concern and urgency for early detection. Men participated mainly because they viewed it as a valuable and convenient opportunity, even if they had not actively considered testing or had no immediate health concerns.

## 4 Discussion

### 4.1 Main findings compared with previous research

This study explored men’s opinions regarding the OPT invitation letters and the attitudes influencing participation decisions. Most found the letters informative but suggested improvements for conciseness and clarity regarding benefits and risks. Participation decisions were influenced by practical considerations, as well as personal and psychological factors.

Our findings align with previous research, indicating that although men value screening opportunities, concerns about the test’s invasiveness and necessity can impede participation [35], [24], [25], [26], [27]. Previous studies have also highlighted that low levels of worry and limited knowledge about PCa can lead to decisions to decline screening. In contrast, a greater perception of the health benefits of screening has been identified as a predictor of participation [36]. This is consistent with our findings, where facilitators to testing included a desire to confirm or rule out PCa and the opportunity to take advantage of available testing. However, some men in our study regretted missing the opportunity due to insufficient prioritisation.

Notably, informants did not weigh the risk-benefit balance when deciding on participation, despite mentioning shortcomings in the provided information about the pros and cons of screening. Instead, practical issues (e.g., lack of time and inaccessibility) and preinvitation concerns (e.g., fear of a cancer diagnosis and distrust) were emphasised. This suggests that men are more influenced by practical and emotional factors than by detailed medical information, aligning with contemporary psychological theories such as dual-process theory and fuzzy-trace theory. While detailed invitation letters about the pros and cons of screening aim to encourage thoughtful deliberation, these theories propose that people often rely on simplified representations for intuitive decisions. Therefore, they suggest selecting need-congruent formats for communicating risks to enhance health care decision-making [37], [38], [39].

In health care, assumptions about necessary communication must be validated by studying the target audience. Understanding men’s perspectives is essential for tailoring communication and decision-making processes in screening programmes. Despite numerous screening trials, little is known about men’s attitudes towards organised testing. Our study addresses this gap, emphasising the need to listen to their concerns and improve communication. Grasping these attitudes is crucial for enhancing informed decision-making and optimising future screening programmes.

### 4.2 Potential clinical strategies to support informed decision-making

Fig 2 presents potential clinical strategies to support informed decision-making regarding OPT participation. Achieving high participation remains challenging, especially among underrepresented groups. Research on colorectal cancer screening shows that younger men have notably low participation rates [40]. The COM-B model, focusing on “capability”, “opportunity”, and “motivation”, provides a framework for understanding the determinants of desired behaviour and can be useful for supporting informed decision-making [41].

Capability encompasses an individual’s psychological and physical ability to engage in screening. In our study, informants highlighted factors such as understanding the PCa screening process and responding to the invitation. While many found the invitation letters informative, some felt that these lacked clarity on testing risks and benefits. Enhancing participants’ capability could involve providing clearer, more targeted information, which may require further studies to refine the invitation content. Technical barriers, such as difficulties using QR codes, also limited access to information and reduced overall capability to engage in the screening process.

Opportunities include physical and social factors affecting behaviour, such as resource access and environmental conditions. One identified barrier was the inaccessibility of testing sites. Men living far from facilities or with busy schedules struggled to participate despite their interest. Improving logistics, such as offering more convenient locations or extended hours, could enhance physical opportunity and increase participation. This aligns with cervical cancer screening research, which shows that simplifying testing opportunities can boost participation rates [42]. Social influences were also relevant. Although we did not directly assess family or peer support, prior research indicates that encouragement from social networks and health care providers promotes participation [24], [25]. If the Swedish National Board of Health and Welfare revises its current advisories, updated guidance from health care providers could improve social opportunity, clarify the pros and cons of testing, and encourage shared decision-making.

Motivation drives behaviour through both reflective (conscious decisions) and automatic (emotional responses and habits) processes. The primary facilitator for OPT participation was the desire to confirm or rule out PCa, especially among men with personal or family cancer experiences, aligning with previous research on screening reassurance [43]. However, some men deprioritised testing due to competing priorities. Enhancing reflective motivation could involve emphasising the benefits of early detection, targeting low-participation areas, and implementing community-based interventions, as shown in research on colorectal, breast, and cervical cancer screening to increase participation rates [44]. Additionally, fear and anxiety, driving automatic motivation, led many to avoid testing to escape the stress of a potential cancer diagnosis. Addressing these fears through reassurance and normalising the screening process could enhance automatic motivation.

### 4.3 Strengths and limitations

A key strength of our study is its focus on the target populations, valuing their perspectives as expert insights into the OPT invitation process. By engaging both participants and non-participants, we aimed to identify barriers and facilitators to participation, essential for refining PCa screening recommendations and improving implementation. While the sample size may be considered small, it aligns with qualitative study design recommendations, allowing for in-depth interviews and detailed insights [31], [32].

The use of semi-structured interviews and an abductive thematic analysis enabled a thorough exploration of anticipated and emergent themes. This method helped categorise informants by testing status and identify key themes from their responses. Combining deductive categorisation with inductive theme generation provided a comprehensive understanding of decision-making influences, highlighting the areas for improvement in screening communication and the need to address barriers to support informed decision-making.

The study has limitations. Its single-county focus may limit the transferability of our findings. A recall bias might affect informants’ reflections, as interviews occurred 2 months after the OPT invitation. The focus on men aged 50 years may not capture the attitudes of older men at risk of PCa. Finally, urologists in the coding team might have introduced a bias due to their presumed opinions and familiarity with the context. To mitigate this and ensure reliability, we employed research triangulation with a diverse team of varying expertise for both interviews and analysis, comparing results interactively with informants’ quotations.

## 5 Conclusions

This study provides valuable insights into men’s attitudes towards PCa screening through OPT and identifies factors influencing participation. By exploring both participants’ and non-participants’ perspectives, we identified practical considerations, as well as personal and psychological factors influencing participation, along with the need for clear communication. Future recommendations should consider these insights to better align screening practices with the target population’s needs and concerns.

  ***Author contributions*:** Markus Arvendell had full access to all the data in the study and takes responsibility for the integrity of the data and the accuracy of the data analysis.

  *Study concept and design*: Arvendell, Phillips, Delilovic, Bolejko, Richter, Lantz.

*Acquisition of data*: Arvendell, Backman Enelius, Olsson.

*Analysis and interpretation of data*: Arvendell, Phillips, Bolejko, Lantz.

*Drafting of the manuscript*: Arvendell.

*Critical revision of the manuscript for important intellectual content*: All authors.

*Statistical analysis*: Arvendell, Phillips, Bolejko, Lantz.

*Obtaining funding*: Arvendell, Lantz.

*Administrative, technical, or material support*: None.

*Supervision*: Richter, Lantz.

*Other*: None.

  ***Financial disclosures:*** Markus Arvendell certifies that all conflicts of interest, including specific financial interests and relationships and affiliations relevant to the subject matter or materials discussed in the manuscript (e.g., employment/affiliation, grants or funding, consultancies, honoraria, stock ownership or options, expert testimony, royalties, or patents filed, received, or pending), are the following: None.

  ***Funding/Support and role of the sponsor*:** None.
